# Supplementary material for: Effects of Sodium Nitroprusside on Lipopolysaccharide-Induced Inflammation and Disruption of Blood–Brain Barrier
Source: Cells. 2024 May 15;13(10):843. doi: 10.3390/cells13100843 (PMC11119468; doi:10.3390/cells13100843)
Supplement: Supplementary file 1 [file cells-13-00843-s001.zip › cells-2995716-supplementary.pdf]

Supplementary Materials

Effects of sodium nitroprusside on LPS-induced inflammation and disruption of blood-brain barrier

Nuria Seoane[1], Aitor Picos[1], Sandra Moraña-Fernández[1,2], Martina Schmidt[3], Amalia Dolga[3], Manuel Campos-Toimil[1,4*$], Dolores Viña[1,4$]

1. Physiology and Pharmacology of Chronic Diseases (FIFAEC) Center for Research in Molecular Medicine and Chronic Diseases (CiMUS), University of Santiago de Compostela, Santiago de Compostela, Spain; nu-ria.seoane@rai.usc.es; aitor.picos@rai.usc.es; manuel.campos@usc.es; mdolores.vina@usc.es
2. Present address: Cardiology Group, Center for Research in Molecular Medicine and Chronic Diseases (CI-MUS) and Institute of Biomedical Research of Santiago de Compostela (IDIS-SERGAS). Av. Barcelona, Campus Vida, 15782 Santiago de Compostela, Spain; sandra.morana.fernandez@usc.es
3. Department of Molecular Pharmacology, University of Groningen, Groningen, The Netherlands.; m.schmidt@rug.nl; a.m.dolga@rug.nl
4. Department of Pharmacology, Pharmacy and Pharmaceutical Technology. University of Santiago de Com-postela, 15782 Santiago de Compostela, A Coruña, Spain

* Correspondence: manuel.campos@usc.es

$ Equal contribution

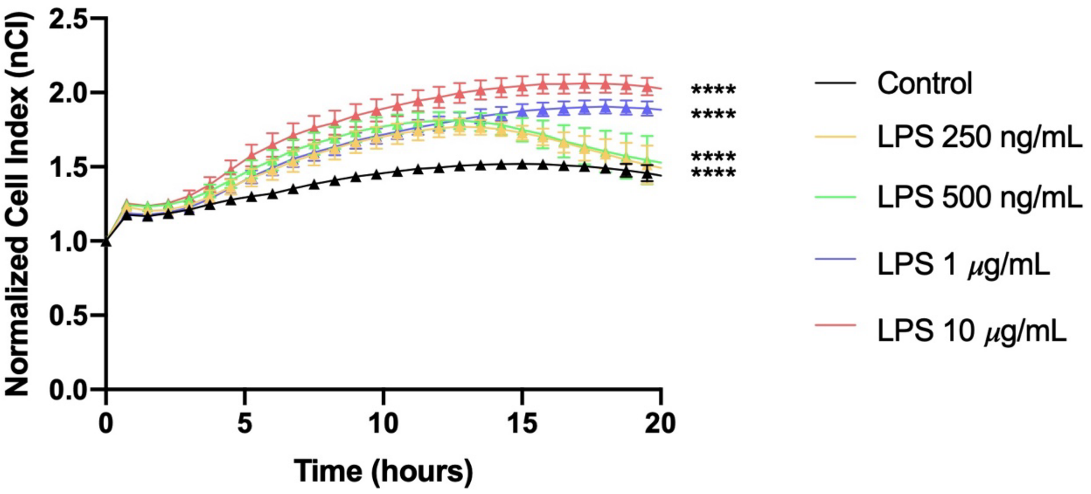

Figure S1. Effect of increasing concentrations of LPS in cellular shape and/or morphology. nCI of bEnd.3 cells exposed to increasing LPS concentrations (0.25, 0.5, 1 and 10 µg/mL) determined by xCELLigence RTCA. Results are given as mean ± SEM; n=5 technical replicates. All experiments were repeated at least four times. ****P<0.0001 with respect to control. Data were analyzed using 1-way ANOVA followed by Sidák's multiple comparisons test.

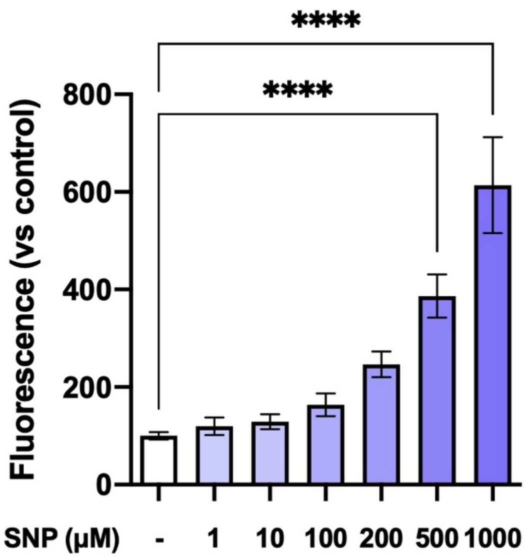

Figure S2. Effect of increasing concentrations of SNP in ROS production. Quantitative determination of ROS production of bEnd.3 cells exposed to increasing SNP concentrations. Results are given as mean ± SEM; n=3 technical replicates. All experiments were repeated at least three times. ****P<0.0001 vs control. Data were analyzed using 1-way ANOVA followed by Sidák's multiple comparisons test.

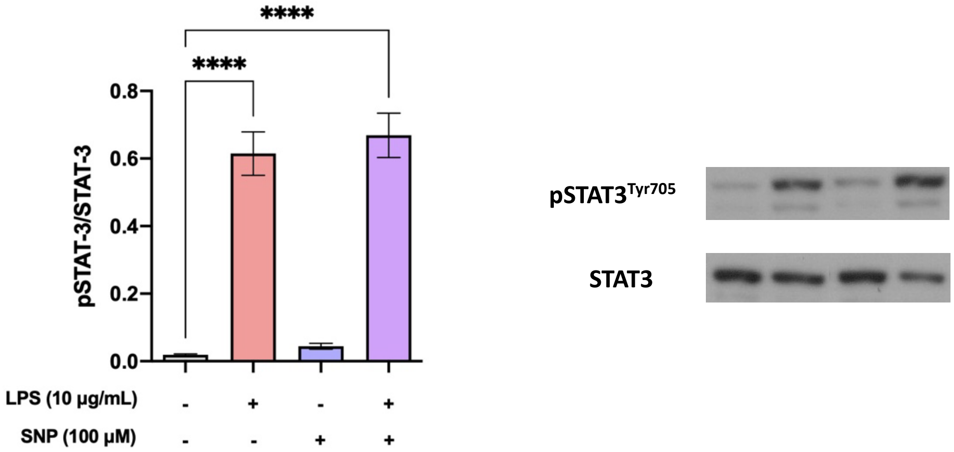

Figure S3. Effect of SNP on LPS-induced STAT3 phosphorylation. STAT3 phosphorylation analyzed by Western Blot after 6-hour exposure to LPS in the presence or absence of SNP. Results are given as mean ± SD. Experiments were repeated at least four times. ****P<0.0001 vs as indicated in the graph. Data were analyzed using 1-way ANOVA followed by Sidák's multiple comparisons test.
